# TCF 4 tumor suppressor: a molecular target in the prognosis of sporadic colorectal cancer in humans

**DOI:** 10.1186/s11658-020-00217-w

**Published:** 2020-03-31

**Authors:** Mumtaz Anwar, Pooja Malhotra, Rakesh Kochhar, Alka Bhatia, Akhtar Mahmood, Rajinder Singh, Safrun Mahmood

**Affiliations:** 1 Department of Experimental Medicine and Biotechnology, PGIMER, Chandigarh, 160012 India; 2 Department of Gastroenterology, Postgraduate Institute of Medical Education and Research, Chandigarh, 160012 India; 3 Department of Pharmacology, University of Illinois at Chicago, Chicago, 60612 USA; 4 Department of Medicine, University of Illinois at Chicago, Chicago, 60612 USA; 5 Department of Biochemistry, Panjab University, Chandigarh, 160014 India; 6 Department of Surgery, Postgraduate Institute of Medical Education and Research, Chandigarh, 160012 India

**Keywords:** TCF-4, Genetic alterations, Quantitative real-time PCR, IHC, Confocal microscopy, Western blotting, Clinicopathological factors

## Abstract

**Background:**

A huge array of function is played by the Wnt/β-catenin signaling pathway in development by balancing gene expression through the modulation of cell-specific DNA binding downstream effectors such as T-cell factor/lymphoid enhancer factor (TCF/LEF). The β-catenin/TCF-4 complex is a central regulatory switch for differentiation and proliferation of intestinal cells (both normal and malignant). Thus, in the present study we evaluated each of 60 cases of sporadic adenocarcinoma, alongside adjoining and normal mucosa specimens of colorectum in humans, for mutation and expression analysis of the gene coding for TCF-4 protein.

**Methods:**

DNA sequencing following PCR amplification and SSCP analysis (single strand conformation polymorphism) was employed to detect TCF-4 gene mutations in the case of exon 1. Quantitative real-time (qRT) PCR, immunohistochemistry (IHC), confocal microscopy and western blot analysis were used to detect TCF-4 gene/protein expression.

**Results:**

Sequencing analysis confirmed 5/60 patients with a point mutation in exon 1 of the TCF-4 gene in tumor samples. mRNA expression using qRT-PCR showed approximately 83% decreased TCF-4 mRNA expression in tumor tissue and adjoining mucosa compared to normal mucosa. Similarly, a significant decrease in protein expression using IHC showed decreased TCF-4 protein expression in tumor tissue and adjoining mucosa compared to normal mucosa, which also corresponds to some important clinicopathological factors, including disease metastasis and tumor grade. Mutational alterations and downregulation of *TCF-4* mRNA and hence decreased expression of TCF-4 protein in tumors suggest its involvement in the pathogenesis of CRC.

**Conclusions:**

A remarkable decrease in TCF-4 mRNA and protein expression was detected in tumorous and adjoining tissues compared to normal mucosa. Hence the alterations in genomic architecture along with downregulation of *TCF-4* mRNA and decreased expression of TCF-4 protein in tumors, which is in accordance with clinical features, suggest its involvement in the pathogenesis of CRC. Thus, deregulation and collaboration of TCF-4 with CRC could be a concrete and distinctive feature in the prognosis of the disease at an early stage of development.

## Novelty and impact statement

TCF-4, a tumor suppressor and a DNA binding factor, which is a cell-specific key downstream effector of the Wnt/β-catenin pathway, upon modulation causes a critical event in colorectal cancer (CRC) development in humans. Decreased mRNA and protein are probably due to alterations in genomic architecture in tumorous tissue along with some specific clinicopathological characteristics that may demonstrate a decisive event in CRC development, and it could be also an early event as a prognostic factor in the development of sporadic CRC in humans.

## Background

Colorectal cancer is one of the leading causes of death worldwide and it is the 2nd most common in women and 3rd most prevalent cancer in men. The Wingless-type (Wnt) signaling pathway plays an important role in embryonic development, and dysregulation of this pathway leads to pathological conditions such as cancer [1]. Moreover, the role of Wnt signaling pathway in colorectal carcinogenesis is well established [2]. A key component of the Wnt signaling pathway is the adenomatous polyposis coli (APC) gene. APC is a tumor suppressor gene which is found to be mutated in the majority of familial adenomatous polyposis (FAP) and sporadic colorectal cancers (CRCs) [3, 4]. The Wnt signaling pathway is of two types: β-catenin dependent (canonical) and independent (non-canonical). In the canonical pathway, in the absence of Wnt ligands, APC along with casein kinase (CK1α) and glycogen synthase kinase (GSK)-3β forms a destruction complex around the β-catenin and degrades β-catenin via the ubiquitin proteasomal degradation pathway [5, 6]. This causes histone deacetylases (HDACs) or other co-repressors to bind to the transcription factor TCF in the nucleus, causing transcription repression of Wnt target genes [7]. In CRC patients there is a homozygous loss of the APC gene which prevents the destruction of β-catenin. As result, there is accumulation of β-catenin in the nucleus, which leads to transactivation of TCF by displacing co-repressors and recruiting co-activators, causing increased transcription of Wnt responsive genes mediating proliferation and invasion.

TCF is a transcription factor of the T cell family which binds to β-catenin and regulates transcription of Wnt target genes [8]. In vertebrates there are five TCF genes and each TCF has different functions. All of these TCF/LEF transcription factors are downstream cell-specific effectors of the wnt/β-catenin signaling pathway that contain a β-catenin binding domain at the N-terminus and a DNA-binding HMG (high mobility group) box [9]. Binding of various co-activators and co-repressors determines the transcriptional activity of these factors.

Dysregulation of the Wnt/β-catenin signaling pathway results from mutation in the APC gene leading to hyper-proliferation required for the initiation and progression of colorectal cancer. TCF-4 is the binding partner for β-catenin in the colon which mediates the effects of hypersignaling activity of β-catenin by stimulating a variety of gene promoters in the nucleus thus resulting in proliferation, differentiation, epithelial-mesenchymal transition and neoplastic progression [10, 11]. In addition, it has been shown that TCF-4 plays an important role in maintaining the stem cells of the crypt in the gut epithelium as TCF-4^−/−^ mice have fewer differentiated villi that lack crypt stem cells.

Previous studies did not reveal any mutations in exon 1 of the TCF-4 gene in the case of renal cell carcinoma. However, Duval A et al. [12] reported a 2-bp deletion at coding position 112–115 in the GAGA nucleotides within exon 1 in the LS-1034 colon cancer cell line that impairs the function of TCF-4. Furthermore, it has been reported that the progenitor cell proliferation may require TCF-4, and the loss of the proliferative compartment of the cell could result due to the loss of TCF-4. Thus, TCF-4 could be an important factor in regulating proliferation and loss of its expression may lead to persistent proliferation [11, 13]. Dysregulation of TCF-4 transcription factor in the development of esophageal squamous cell carcinoma as well as in the colorectal carcinogenesis has been shown previously. However, whether dysregulation of TCF-4 plays a role in the development of colorectal carcinogenesis in the Indian population is not known.

Therefore, the present study investigated the mutational analysis of exon 1 of the TCF-4 gene and the expression analysis of TCF-4 in sporadic colorectal cancer patients in a north Indian population. Our findings demonstrated for the first time the presence of mutations in exon 1 along with decreased expression of TCF-4 gene/protein in tumor tissues compared to normal and adjoining mucosa.

## Materials and methods

### Patients and specimens

Specimens were collected from 60 sporadic colorectal adenocarcinoma patients who underwent surgery at the Postgraduate Institute of Medical Education and Research, India. The current study was designed to examine the clinicopathological features of the CRC patient as well as the mutation and expression pattern of transcription factor 4 (*TCF-4*) in tumor, adjoining and normal colorectal tissues. There were 40 male and 20 female patients. The age of the patients ranged from 21 to 82 years and median age was 52 years. Informed consent was obtained from each patient before taking the sample. The study was approved and authorized by the Institute Ethics Committee. The colon and rectum were also examined for the presence of any tumor or synchronous polyp. Tumor, adjoining (2.5 cm distant from tumor) and normal mucosa (5–10 cm distant from tumor) samples were collected from a fresh colorectal resected specimen. All the specimens were divided into two equal halves. One half was snap frozen in liquid nitrogen and then transferred to − 80 °C. The other half was fixed instantly in 10% formalin for 24 h, paraffin embedded and H&E staining was done to determine the grade and tumor invasion [14]. Tumor stages were defined according to the TNM classification [15].

### DNA extraction and PCR amplification of exon 1 of *TCF4* gene

DNA was isolated from all the specimens using phenol/chloroform extraction [16]. PCR analysis of exon 1 of the TCF-4 gene was performed using gene specific primers 5′-AATTGCTGCTGGTGGGTGA-3′ (sense) and 5′-CCCGAGGGGCTTTTCCTA-3′ (antisense). An amplicon of 234 bp was obtained after PCR amplification.

### Single strand conformation polymorphism (SSCP) analysis

PCR-SSCP analysis was used to “prescreen” the samples that contain mutations in the TCF4 gene. Amplified PCR products of exon 1 of the TCF4 gene (7 μl) were diluted with equal amounts of denaturing buffer and kept at 95 °C for 5 min, and instantly cooled down on ice. Samples were then run on a non-denaturing polyacrylamide gel (10%) at 60–80 V at 4 °C for 25–30 h. Gels were stained using EtBr staining as described previously [5, 17].

### DNA sequencing

DNA samples with suspected mutations (as determined by SSCP analysis) in exon 1 of the *TCF-4* gene were amplified deploying Phusion polymerase enzyme (high fidelity) (New England Biolabs, Finland). Sequencing was done using an automated DNA Sequencer (ABI 3730XL Genetic analyzer; Xcelris Genomics). All sequences obtained were aligned with sequences previously published in NCBI for exon 1 of the TCF4 gene to find the mutation.

### Reverse transcription (quantitative real-time) PCR

TCF-4 mRNA levels were studied using quantitative real-time PCR. The Trizol method was used to isolate RNA from tumor, adjoining and normal mucosa [18]. Two micrograms of RNA was used to carry out reverse transcription using a cDNA synthesis kit (Applied Biosystems). cDNA was then further amplified by PCR using primers specific for the human TCF-4 gene; forward 5′-AAAGCGCGGCCATCAAC-3′ and reverse 5′- CAGCTCGTAGTATTTCGCTTGCT-3′. Primers for β-actin were: forward 5′- CCTGTACGCCAACACAGTGC-3′ and reverse 5′-ATACTCCTGCTTGCTGATCC-3′. The amplification reaction comprised of various steps: denaturation at 94 °C for 40 s, annealing at 58 °C for 30 s, and extension at 70 °C for 60 s for 35 cycles. The 2^-Δct^ method was used to analyze q-PCR results.

### Immunohistochemistry

IHC analysis was performed by the streptavidin-biotin peroxidase complex method using paraffin-embedded tissue sections. Sections were deparaffinized at 65 °C in an oven and rehydrated followed by antigen retrieval (citrate buffer 10 mM (pH 6.0) for 10 min at 100 °C in a microwave oven). For IHC staining, TCF-4 Orb108692 primary antibody was used to detect TCF-4 protein (Biorbyt, USA). After the primary antibody incubation sections were washed with buffer and then incubated with secondary antibody for 1 h at room temperature. Sections were subsequently washed with buffer and then mounted with Prolong gold antifade mountant. A score of 0–3 for staining intensity was assigned as described by Miyamoto et al. [19]. An intensity score of 0 = no staining, 1 = weak positivity, 2 = moderate positivity and 3 = strong positivity was given. The method mentioned below was used to calculate IHC score = %age of positivity x intensity score. Images were taken using light microscopy. Similar parameters were employed to analyze the results of confocal microscopic images. All these results were analyzed in a blinded way.

### Confocal microscopy

The protocol for confocal microscopy was similar to that of IHC analysis until primary antibody incubation. After primary antibody incubation, slides were washed with buffer and then stained with Alexa flour 488 secondary antibody incubation for 40 min in a dark room at a dilution of 1:500. After that sections were counterstained with Hoechst stain for 10 min and visualized under a laser confocal microscope (MRC 600; Bio-Rad/Analytical Instr. Group, Cambridge, MA), using a 63X Zeiss aim-plan oil immersion objective (NA 1.4). Images acquired on the green channel were photographed from the computer screen. Confocal images were scored, and intensity was calculated as per IHC scoring analysis of this study.

### Western blotting

Total proteins were isolated from all the different tissues samples using a homogenizer and RIPA lysis buffer (Cell Signaling, Danvers, MA). Protein concentration was determined using the Bradford method. To examine the expression of TCF4 protein equal amounts of proteins from tumor, adjoining and normal tissues were run on SDS-polyacrylamide gel. The method for western blotting was followed as described by Mahmood et al. [20]. Membrane was kept in blocking solution and then incubated with primary antibody (1:500 v/v dilution, rabbit polyclonal anti-human TCF-4 [Orb108692], Biorbyt, USA) for 2–4 h followed by incubation with secondary antibody which is anti-rabbit IgG (HRP) in goat (1:1000 v/v dilution). β actin was used as a loading control. The immuno-blots were developed using the DAB system and the reaction was stopped by the addition of excess water [5, 20]. Imaging of TCF4 and actin blots were done in the Alpha Innotec gel documentation system (Fisher Scientific, USA) and densitometric analysis of the blots was done using image J software.

### Statistical analysis

The relationship between mutation pattern and their respective protein expression was determined by Pearson’s χ^2^ test. The relationship between TCF-4 expression and each of the clinicopathological parameters was determined by the Mann-Whitney U test. ANOVA followed by post-hoc test was performed for the comparisons of different groups. The Kaplan-Meier method was employed to evaluate the disease-free (DFS) survival and overall survival (OS) of patients and the log rank test was used to evaluate the difference in survival of patients.

## Results

### Mutation detection in exon 1 of TCF-4 gene by PCR amplification and SSCP analysis

**Fig. 1:**
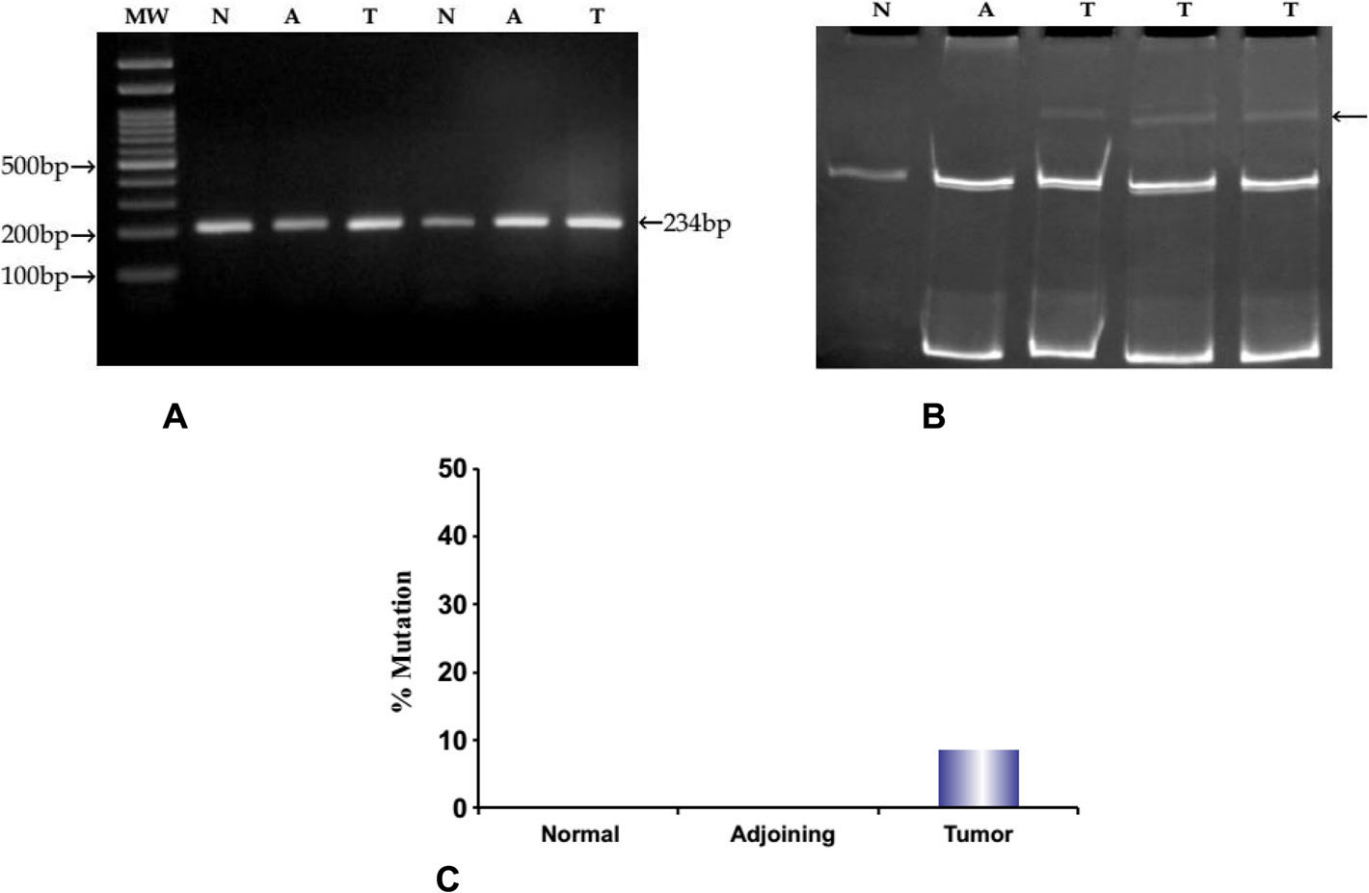
Mutation detection in exon 1 of TCF-4 gene by PCR amplification and SSCP analysis. **a** shows agarose gel electrophoresis of PCR product of exon 1 of TCF-4 gene from 2 representative cases of CRC (N: normal tissue, A: adjoining tissue, T: tumor tissue). MW: molecular weight marker (100 bp DNA ladder). Arrow represents the amplified PCR product of 234 bp. **b** shows SSCP analysis of PCR product of exon 1 of TCF-4 gene. Gel picture shows the representative image of the three samples that demonstrates the band shift. Arrow represents the bands showing mobility shift in tumor tissues compared to adjoining and normal tissues. **c** shows the percentage of mutations in exon 1 of TCF-4 gene in tumor tissues compared to adjoining and normal tissue

We examined the mutations in exon 1 of the TCF4 gene as it encodes the β-catenin-binding domain. To examine the mutations, PCR-SSCP analysis was performed in all the tumors, adjoining as well as in normal tissues samples from 60 patients using gene-specific primers. Amplified PCR products were run on agarose gel and an amplified product of 234 bp is shown in Fig. 1a. For SSCP analysis of exon 1 of the TCF-4 gene, products of PCR were run on 10% polyacrylamide gel under non-denaturing conditions. A total of 5 cases out of 60 tumor samples showed a band shift in exon 1 of the TCF4 gene, whereas no mobility shift was observed in normal and adjoining samples (Fig. 1b). The mutation frequency calculated from the above results was 8.33% (5/60) for tumor tissue (Fig. 1c).

### DNA sequence analysis of TCF-4 gene

DNA sequence analysis of the 5 tumor samples (S2, S11, S33, S34 and S50) which showed a band shift by SSCP analysis revealed the presence of point mutations in exon 1 of the TCF-4 gene (Table 1). In sample S2, two mutations were observed, one missense and one nonsense at codons 57 and 64 of exon 1 of the TCF-4 gene, which resulted in a change from AAC → ACA (asparagine→threonine) and GTA → TAG (valine → STOP). In sample S11, two mutations were observed, one silent and one nonsense at codons 56 and 64, resulting in alteration of CCA → CAA (glutamine → glutamine) and GTA → TAG (valine → STOP). In sample S33, two mutations were observed, one silent and one nonsense mutation at codons 22 and 41, with a change of AAA → AAG (lysine → lysine) and TTA → TAG (leucine → STOP). In sample S34, three mutations were observed, one silent and two nonsense mutations at codons 37, 41 and 48, which resulted in the alteration of GCA → GCG (alanine → alanine), TTA → TAG (leucine → STOP) and CTA → TAG (leucine → STOP) respectively. Similarly, in sample S50, four mutations were detected, one silent and three nonsense mutations at codons 33, 41, 48 and 64, which resulted in the alteration of GAA → GAA (glutamic acid→glutamic acid), TTA → TAG (leucine→STOP), CTA → TAG (leucine → STOP) and GTA → TAG (valine → STOP) respectively.

**Table 1:** Mutations in Exon 1 of TCF-4 gene in Tumor Samples

Sample	Fragment	Codon	Nucleotide altered	Predicted product
S-2	Exon 1	57	AAC → ACA	Asparagine → Threonine
S-2	Exon 1	64	GTA → TAG	Valine → STOP
S-11	Exon 1	56	CCA → CAA	Glutamine → Glutamine
S-11	Exon 1	64	GTA → TAG	Valine → STOP
S-33	Exon 1	22	AAA → AAG	Lysine → Lysine
S-33	Exon 1	41	TTA → TAG	Leucine → STOP
S-34	Exon 1	37	GCA → GCG	Alanine → Alanine
S-34	Exon 1	41	TTA → TAG	Leucine → STOP
S-34	Exon 1	48	CTA → TAG	Leucine → STOP
S-50	Exon 1	33	GAA → GAA	Glutamic Acid→Glutamic Acid
S-50	Exon 1	41	TTA → TAG	Leucine → STOP
S-50	Exon 1	48	CTA → TAG	Leucine → STOP
S-50	Exon 1	64	GTA → TAG	Valine → STOP

### mRNA expression analysis of TCF-4 gene by qRT-PCR

**Fig. 2:**
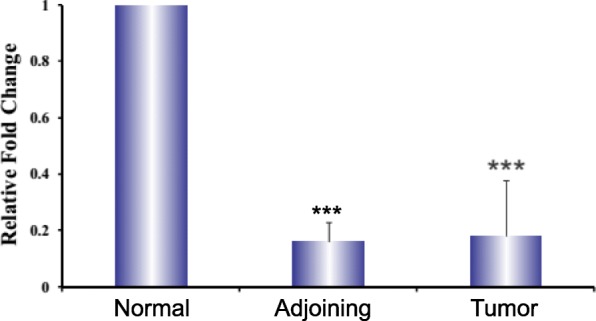
mRNA expression analysis of TCF-4 gene by qRT PCR. Relative mRNA levels of TCF-4 gene in tumor, adjoining and normal mucosa from CRC Patients. ***p < 0.001 compared to normal tissue

Next, we examined the mRNA expression analysis and we observed a (~ 83%) decrease in mRNA expression of the TCF-4 gene in tumors and adjoining tissues as compared to normal mucosa. Using primers specific for the TCF-4 gene and β-actin, we showed that the mRNA expression of TCF-4 gene was decreased significantly (~ 6 fold) in tumors and adjoining tissue as compared to normal tissues (Fig. 2).

### Analysis of TCF-4 protein expression by immunohistochemistry

**Fig. 3:**
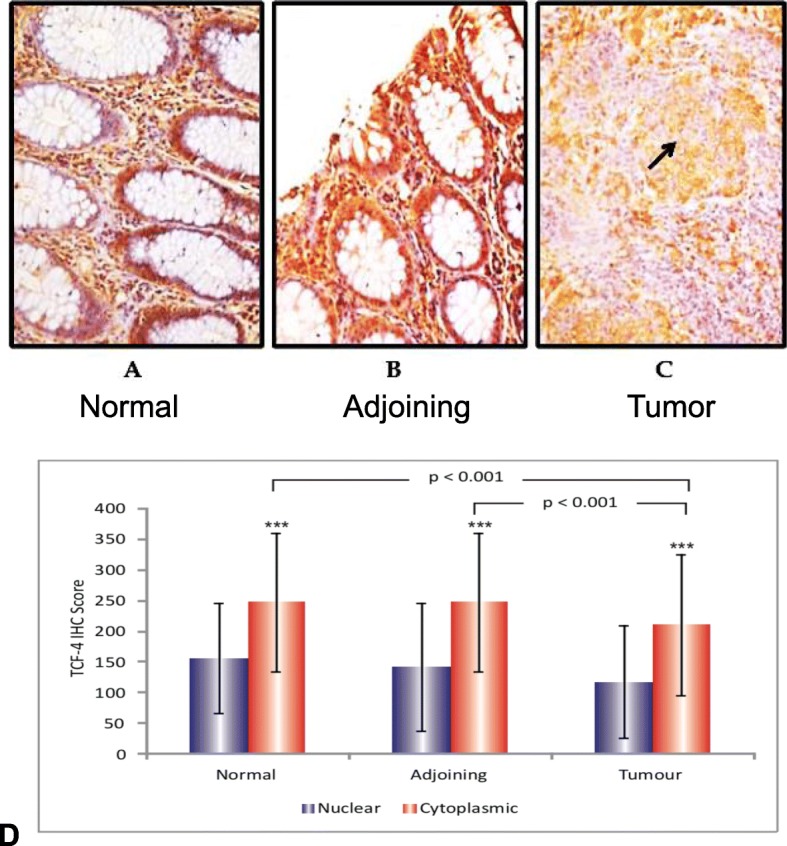
Analysis of TCF-4 protein expression by immunohistochemistry. **a, b** & **c** show immunohistochemical analysis of TCF-4 protein in normal (**a**), adjoining (**b**) and tumor tissues (**c**) from CRC Patients. Magnifications X = 400. **d** shows the nuclear and cytoplasmic immunohistochemical scores of TCF-4 protein in normal, adjoining and tumor tissues from CRC patients

In both normal and adjoining mucosa, protein expression of TCF-4 was present in all the 60 cases analyzed with cytoplasmic expression in 23.3% (14/60), nuclear expression alone in 16.6% (10/60) and both nuclear and cytoplasmic expression in 60% (36/60) of cases studied. The protein expression was strong in 50 and moderate in 10 cases in normal tissues (Fig. 3a). The observed positivity was strong in 50, moderate in 6 and weak in 4 cases in adjoining tissues (Fig. 3b). The TCF-4 expression in normal tissues was high as compared to that in tumor tissues as well as in adjoining mucosa.

However, TCF-4 protein expression was observed in 100% (60/60) of the tumors evaluated with no loss of protein expression. 16.6% (10/60) of samples showed loss of nuclear protein expression. 18.3% (11/60) cases showed cytoplasmic positivity, 16.6% (10/60) cases showed nuclear and 65% (39/60) samples (Table 2) manifested both cytoplasmic and nuclear. The observed downregulation of TCF-4 expression at the protein level in tumor specimens revealed that TCF-4 may act as a tumor suppressor in these subjects. Overall weak expression of the protein was observed in tumor tissues as compared to normal mucosa (Fig. 3c).

**Table 2:** Comparison of Nuclear & Cytoplasmic expression of TCF-4 protein in tumor samples

			TCF-4 (Cytoplasmic) IHC		Total
			NO	YES	
TCF-4 (Nuclear) IHC	**NO**	**Count**	0	11	11
		**% within TCF-4 (Nuclear) IHC**	0.0%	18.3%	18.3%
	**YES**	**Count**	10	39	49
		**% within TCF-4 (Nuclear) IHC**	16.6%	65%	100.0%
Total		**Count**	10	50	60
		**% within TCF-4 (Nuclear) IHC**	16.6%	83.3%	100.0%

Statistically significant variation was observed in the staining of TCF-4 protein in case of tumor (*p* < 0.001), adjoining (*p* < 0.001) and normal mucosa (*p* < 0.001) between the nuclear and cytoplasmic stain (Fig. 3d and Table 3).

**Table 3:** Immunohistochemical scores for TCF-4 protein in tumor, adjoining and normal mucosa of CRC patients

Proteins & Localisation	Range	Normal	Adjoining	Tumor
		Mean ± S.D.	Mean ± S.D.	Mean ± S.D.
TCF-4 IHC (Nuclear)	0–300	156.67 ± 90.416	141.67 ± 103.812	118.00 ± 90.961
TCF-4 IHC (Cytoplasmic)	0–300	247.50 ± 112.153	247.50 ± 112.153	210.83 ± 114.274

The protein expression of β-catenin was observed in all the cases analyzed. Both the nuclear and cytoplasmic β-catenin was higher in the case of tumor tissues as compared to normal mucosa cases (Supplementary Figure 1).

### TCF-4 protein expression by confocal microscopy

**Fig. 4:**
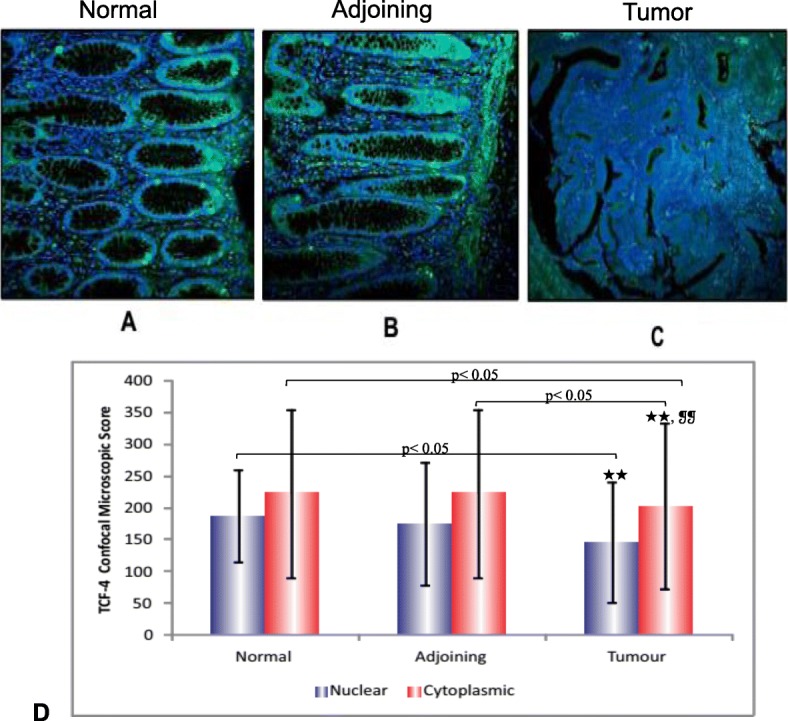
TCF-4 protein expression by confocal microscopy. **a, b** & **c** show confocal immunofluorescent images of TCF-4 protein in normal (**a**), adjoining (**b**) and tumor tissue (**c**) from CRC patients. Magnification X = 200. **d** presents a histogram that shows differential nuclear and cytoplasmic protein expression of TCF-4 protein by confocal microscopy in normal, adjoining and tumor tissues. * indicates *p* value between nuclear protein levels, ❡ indicates *p* values between cytoplasmic protein levels

In normal and adjoining mucosa, intact TCF-4 protein expression was present in all the cases (*n* = 20) having both nuclear and cytoplasmic expression. A representative confocal image of normal (A), adjoining (B) and tumor tissue (C) is shown in Fig. 4. TCF-4 protein was expressed in 100% (20/20) of the tumor tissues analyzed with cytoplasmic positivity in 10% (2/20) of cases, nuclear expression in 25% (5/20) of cases, and 65% (13/20) of cases showed both nuclear and cytoplasmic positivity (Fig. 4d). Overall, weak expression of TCF-4 protein was observed in tumor tissues as compared to normal or adjoining mucosa (Table 4). The observed downregulation of TCF-4 protein in tumor tissues further confirmed the results of immunohistochemistry.

**Table 4:** Confocal microscopic scores for TCF-4 proteins in tumor, adjoining and normal mucosa of CRC patients

Proteins & Localization	Range	Normal	Adjoining	Tumor
		Mean ± S.D.	Mean ± S.D.	Mean ± S.D.
TCF-4 CFM (Nuclear)	0–300	187.50 ± 72.321	175.00 ± 96.655	146.50 ± 94.271
TCF-4 CFM (Cytoplasmic)	0–300	222.50 ± 132.263	222.50 ± 132.263	202.50 ± 130.258

### Western blot analysis of TCF-4 protein

**Fig. 5:**
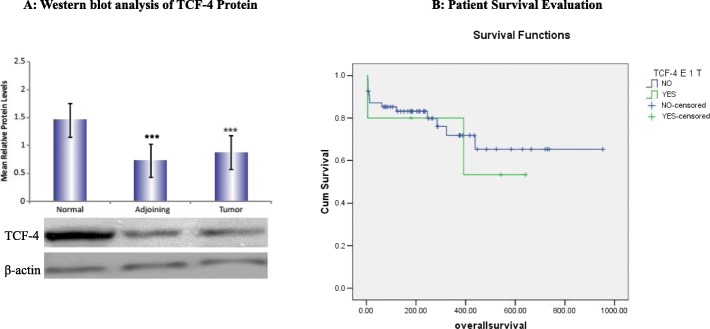
**a** Western blot analysis of TCF-4 protein. Western blot analysis of TCF-4 protein in normal, adjoining and tumor tissues from CRC Patients. Bar diagram showing densitometric analysis of the TCF-4 protein normalized with β-actin protein in normal, adjoining and tumor tissues. Protein levels of TCF-4 were lower in adjoining and tumor tissues of CRC patients compared to normal tissue. Western blot of β-actin as a loading control. ***p < 0.001 compared to normal tissue. A total of 20 cases each of tumor, adjoining and normal mucosa were analyzed for protein expression by western blotting studies. **b** Patient survival evaluation. Kaplan-Meier survival curves were constructed and log rank analysis revealed that TCF-4 exon 1 gene mutations had no correlation with overall survival or disease-free survival of the patients (*p* > 0.05)

Similarly to mRNA expression analysis, the results of the western blot analysis also showed a decrease in the expression of TCF-4 protein both in tumor and adjoining tissues (~ 55%) compared to normal tissues. However, there was no difference in the protein expression of TCF-4 between tumor and adjoining tissues (Fig. 5a). Densitometric analysis showed a significant decrease in the TCF-4 protein expression in tumor and adjoining tissues compared to normal tissues (Fig. 5a).

#### Correlation of clinicopathological factors with TCF-4 mutations

A significant correlation was observed between mutations in exon 1 of the TCF-4 gene and post-operative serum CEA levels (*p* = 0.002) as analyzed by Pearson’s chi-square test (Table 5). However, no correlation was observed with any of these clinicopathological factors such as sex, age, tumor grade, TNM, stage, survival/death, etc. (Table 5).

**Table 5:** Correlation of Clinicopathological factors with TCF-4 Tumor Mutations

Correlations				Correlation Coefficient
		Clinicopathological Factors	Sig. (2-tailed)	
Spearman’s rho	**TCF-4 Tumor Mutations**	**Sex**	***p*** **= 0.**558	***r*** **= −0.183**
		**Age**	***p*** **=** 0.586	***r*** **= −0.078**
		**Tumor grade**	***p*** **=** 0.109	***r*** **= −0.045**
		**Tumor size**	***p*** **=** 0.689	***r*** **= 0.141**
		**TNM stage**	***p*** **=** 0.515	***r*** **= 0.172**
		**Smoking/no smoking**	***p*** **=** 0.787	***r*** **= −0.034**
		**Survival/Death**	***p*** **=** 0.587	***r*** **= 0.100**
		**Recurrence**	***p*** **=** 0.647	***r*** **= 0.100**
		**Serum CEA**	***p*** **= 0.** 0.002	***r*** **= −0.035**

#### Correlation of gene mutation with survival of CRC patients

No correlation of TCF-4 gene mutations was observed with survival (overall and disease-free) of the patients (*p* > 0.05). However, TCF-4 gene mutations in tumors (*p* = 0.040) had a significant correlation with metastasis of the disease (Table 6).

**Table 6:** Effect of Gene mutations on Survival of CRC patients

Correlations				Correlation Coefficient
		Clinicopathological Factors	Sig. (2-tailed)	
Spearman’s rho / Pearsons Chi square	**TCF-4 Tumor Mutations**	**disease free survival and overall survival**	***p*** **> 0.05**	***r*** **= 0.100**
		**Disease metastasis**	***p*** **= 0.04**	***r*** **= 0.1005**

### Effect of TCF-4 mutations on protein levels

Gene mutations and protein levels of TCF-4 were compared and all the attributes are given in Supplementary Table 1. Out of 60 tumor cases, all were positive for TCF-4 protein expression and 5 cases had a mutation in the TCF-4 gene. Besides the presence of mutations in exon 1 of the TCF-4 gene, these mutations had no significant effect on TCF-4 protein expression either at the nuclear or the cytoplasmic level. However, both these mutations and protein levels could be independent factors for the development of CRC (Table 7).

**Table 7:** Effect of Gene mutations on TCF-4 Protein Expression Levels

Correlations				Correlation Coefficient
		Clinicopathological factors	Sig. (2-tailed)	
Spearman’s rho / Pearsons Chi square	**TCF-4 Tumor Mutations**	**Nuclear protein levels**	***p*** **=** 0.426	***r*** **= 0.040**
		**Cytoplasmic protein levels**	***p*** **=** 0.336	***r*** **= 0.130**

### Correlation of clinicopathological factors with protein expression levels

No correlation was observed between nuclear and cytoplasmic TCF-4 protein with clinicopathological factors including sex, age, tumor size, TNM, survival/death, tumor site, etc., whereas there was a significant correlation between TCF-4 protein levels and tumor grade (*p* = 0.029) [Table 8].

**Table 8:** Correlation of Clinicopathological factors with Protein Expression Levels

Correlations				Correlation Coefficient
		Clinicopathological Factors	Sig. (2-tailed)	
Pearson’s chi square	**TCF-4 Protein Expression Levels**	**Sex**	***p*** **= 0.**912	***r*** **= −0.235**
		**Age**	***p*** **= 0.254**	***r*** **= − 0.022**
		**Tumor site**	***p*** **= 0.360**	***r*** **= 0.098**
		**Tumor size**	***p*** **= 0.864**	***r*** **= 0.142**
		**TNM stage**	***p*** **= 0.924**	***r*** **= −0.044**
		**Survival/Death**	***p*** **= 0.334**	***r*** **= 0.066**
		**Serum CEA**	***p*** **= 0.890**	***r*** **= −0.095**
		**Tumor grade**	***p*** **= 0.029**	***r*** **= 0.025**

### Patient survival evaluation

The survival (disease-free and overall) was analyzed in association with TCF-4 mutations and its expression levels. The study period ranges between 8 and 39 months (mean = 25.5 ± 8.14) and the median follow-up time was 27 months. No correlation of TCF-4 gene mutations and protein expression levels was observed with overall survival or disease-free survival of the patients (*p* > 0.05) (Fig. 5b). However, tumor stage (*p* = 0.001) and its grade (*p* = 0.029) had a significant correlation with disease metastasis. Furthermore, a significant correlation of mutations in adjoining mucosa of the TCF-4 gene (*p* < 0.001) was observed with disease metastasis.

## Discussion

The TCF-4 gene plays an important role in malignant transformation and it forms an important component of the Wnt signaling pathway [8, 21]. TCF-4 shares sequence homology with the HMG box and is a member of the transcriptional factor family [22]. The TCF-4 genomic structure comprises various important domains, i.e. β-catenin-binding, DNA-binding HMG box, and COOH terminal domains in exons 1, 10 and 11, and exon 17, respectively [22]. Previous studies reported high frequency of mutations (10%) in exon 1 of the TCF-4 gene in colorectal cancer compared to other exons (2–3%) [23].

In the current study, mutational analysis of the TCF-4 gene for exon 1 revealed the presence of point mutations in 5 tumor samples, with a frequency of 8.33%, whereas no mutations were observed in adjoining and normal mucosa of exon 1 of the TCF-4 gene. The mutations were missense, nonsense or silent type as described in Table 6 of the Results section. Shiina et al. [22] demonstrated no mutations in exon 1 but found one SNP (proline to threonine) in exon 17 in renal cell carcinoma. Duval et al. [12] observed a frame shift variant with deletion of 2 bp in TCF-4 exon 1 that corresponds to the β-catenin binding domain using the cell line LS 1034, which is consistent with the current study. The COSMIC database (COSMIC v69 Release (2nd June 2014) also reported a frequency of 1.80% for TCF-4 mutations. Different mutational frequencies for the TCF-4 gene were observed in various studies [23]. While we observed mutations in exon 1 of the TCF-4 gene, other studies have found it in different exons, e.g. in exons 4, 3–9 and 17 [12, 24]. So, ours is the first study to report such subtypes of mutations as missense, silent and nonsense in exon 1 of the TCF-4 gene. Further analysis of TCF-4 gene mutations revealed no correlation with clinicopathological factors except with postoperative serum CEA levels (*p* = 0.002) of the patients, as described in the Results section. Jiang Y et al. [25] also found no correlation of mutations with patients’ clinicopathological factors in hepatocellular carcinoma, which is consistent with our findings.

In normal colonic tissue, TCF4 is the dynamically most expressed component of the TCF/LEF gene family [26]. In the present study, a significant decrease in mRNA expression of the TCF-4 gene was observed in tumors and adjoining mucosa as compared to normal mucosa. Although high levels of TCF4 mRNA expression were detected in NSCLC (non-small cell lung cancer) tissue samples, no detectable expression of TCF-4 mRNA was reported in normal lung tissue [27]. In normal renal tissues while employing northern blot and RT-PCR analysis, no TCF-4 mRNA was observed in humans, as reported by Lin G et al. [28]. Similarly, expression of TCF-4 was undetectable in mice normal tissue besides the brain as reported by Korinek V et al. [29]. TCF-4 overexpression has been documented in renal cell [28], hepatocellular [30] and mammary gland carcinoma. However, Angus-Hill ML et al. [9] supported the tumor suppressive function of TCF-4 in colon cells, which is in accordance with our study. Indeed, Cuilliere-Dartigues et al. [26] recorded elevated levels of mRNA expression of TCF-4 in three colorectal cell lines (HCT116, CO115 and LoVo). These observations are contradictory with our results, which is probably due to the heterogeneous pattern of the population and availability of various transcripts for the TCF-4 gene. In the current studies, we also carried out c-*Myc* mRNA expression levels in normal, adjoining and tumor tissues, as c-*Myc* is one of the well-known targets of *β-catenin/TCF4*, and we found that the expression of *c-Myc* was increased in tumor tissues as compared to normal mucosa *(****data not shown****).* Yochum et al. [31] also showed, by using the human HCT116 cell line as a model, that TCF4/β-catenin complexes assembled at the MYC 3′ enhancer and coordinated a chromatin loop with the MYC proximal promoter to activate MYC expression, which is in accordance with our studies [31–33].

TCF-4 protein was expressed in all the tumor tissues and in all the adjoining and normal mucosa samples examined. However, the majority of tumors which expressed TCF-4 protein were low expressers or had reduced expression of TCF-4 protein, which suggested that the expression of TCF-4 protein was significantly reduced during transition from normal epithelium to carcinoma. Confocal microscopy and western blot analysis also confirmed the results of immunohistochemistry, indicating reduced expression of TCF-4 protein in tumor tissues as compared to normal and adjoining mucosa of CRC patients.

In the current report, the TCF-4 protein expression was revealed both in mutation positive and negative cases, and no significant correlation was demonstrated between mutations and TCF-4 protein levels. Such discrepancies might be due to the variability of the gene region explored. If we had evaluated the exon 11 mutations in addition to exon 1 of the TCF-4 gene, a higher probability of mutational frequency could have been expected, as it contains a high-mobility group DNA-binding domain (HMG DBD) [34]. The second reason could be the heterogeneity in the evaluation of protein scoring methods in the current report. As in some earlier studies, immunostaining intensity was considered, but in the present report, both the % age positivity and intensity were considered. Furthermore, in the present study, we did not observe any correlation of TCF-4 protein expression with clinicopathological factors such as age, sex and stage of the disease while a significant correlation of protein expression levels was observed with tumor grade (*p* = 0.029). Takeda K et al. [35] and others [36] have found a significant correlation of expression between TCF4 and β-catenin (*p* = 0.01), whereas they found no association of TCF-4 protein with pT and pN stage.

In the present study, there might be variability between the mRNA and protein expression levels and between the results of IHC, confocal and western blot analysis. Importantly, the stability of mRNA varies as compared to that of protein levels. However, recent studies have quantified transcript and the protein expression levels that revealed the importance of various processes which contribute to establishing the protein expression levels [37].

These processes include 1) translation rates which are directly influenced by sequences of mRNA; 2) modulation of the translation rate, which occurs through the binding of regulatory elements to proteins on transcripts or the availability of the transcripts; 3) half-life of proteins and the role of proteasome ubiquitin pathways or the role of other processes such as autophagy that influences the concentration/amount of protein, which is independent of levels of the transcript, i.e. mRNA levels; 4) changes in the transcripts and delays in the synthesis of proteins will definitely affect the levels and expression of the proteins. Thus, it suggests that the comparison between mRNA and protein for the same cell/tissue/location may be heterogenous [37, 38].

## Conclusion

In conclusion, the alterations in genomic architecture [39, 40] along with significant downregulation of *TCF-4* mRNA and hence decreased expression of TCF-4 protein in tumors in accordance with some specific clinical features suggest its relationship with the pathogenesis of CRC. The deregulatory TCF-4 expression levels could be only an ancillary phenomenon with a functional effect on target gene expression. Thus, deregulation of TCF-4 in CRC subjects from the north Indian population could be suggestive of a distinctive prognostic feature at an early stage of disease development.

## Supplementary information

**Additional file 1.** (PPTX 36670 kb).

**Additional file 2.**

## Data Availability

All data generated or analyzed during this study are included in this published article and its supplementary information files. Some of the mutations presented in this manuscript are novel and a few are in accordance with the previous studies.

## Abbreviations

TCF-4: T cell factor
LEF: Lymphoid enhancer factor
PCR-SSCP: Polymerase chain reaction – single strand conformation polymorphism
CRC: Colorectal cancer
IHC: Immunohistochemistry
HMG: High mobility group box
OS: Overall survival
DFS: Disease-free survival
qRT-PCR: Quantitative real-time polymerase chain reaction
APC: Adenomatous polyposis coli
FAP: Familial adenomatous polyposis
CK1α: Casein kinase
GSK-3β: Glycogen synthase kinase
HDACs: Histone deacetylases
NSCLC: Non-small cell lung cancer

## References

[1] Yang M, Wang M, Li X, Xie Y, Xia X, Tian J (2018). Wnt signaling in cervical cancer?. J Cancer.

[2] Zhan T, Rindtorff N, Boutros M (2017). Wnt signaling in cancer. Oncogene..

[3] Malhotra P, Anwar M, Nanda N, Kochhar R, Wig JD, Vaiphei K (2013). Alterations in K-ras, APC and p53-multiple genetic pathway in colorectal cancer among Indians. Tumour Biol.

[4] Anwar M, Kochhar R, Rather S, Bhatia A, Singh R, Vaiphei K, Mahmood S (2013). Mutations & expression of APC & β-catenin in sporadic colorectal tumors: a mutational “hotspot” for tumorigenesis. J Gastroenterol Hepatol.

[5] Anwar M, Kochhar R, Singh R, Bhatia A, Vaiphei K, Mahmood A, Mahmood S (2016). Frequent activation of the β-catenin gene in sporadic colorectal carcinomas: a mutational & expression analysis. Mol Carcinog.

[6] Anwar M, Bhatia A, Mahmood S (2016). GSK-3β deregulation and mutational alteration in sporadic colorectal Cancer in Indian cohorts. Gastroenterology.

[7] Malhotra P, Anwar M, Kochhar R, Ahmad S, Vaiphei K, Mahmood S (2014). Promoter methylation and immunohistochemical expression of hMLH1 and hMSH2 in sporadic colorectal cancer: a study from India. Tumour Biol.

[8] Anwar M, Kochhar R, Bhatia A, Singh R, Mahmood S (2017). Expression and mutational analysis of Exon 17 of TCF4 transcription factor in sporadic colorectal cancer. Ann Oncol.

[9] Angus-Hill ML, Elbert KM, Hidalgo J, Capecchi MR (2011). T-cell factor 4 functions as a tumor suppressor whose disruption modulates colon cell proliferation and tumorigenesis. Proc Natl Acad Sci U S A.

[10] Vogelstein B, Fearon ER, Hamilton SR, Kern SE, Preisinger AC, Leppert M, Nakamura Y, White R, Smits AM, Bos JL (1988). Genetic alterations during colorectal-tumor development. N Engl J Med.

[11] van de Wetering M, Sancho E, Verweij C, de Lau W, Oving I, Hurlstone A, van der Horn K, Batlle E, Coudreuse D, Haramis AP, Tjon-Pon-Fong M, Moerer P, van den Born M, Soete G, Pals S, Eilers M, Medema R, Clevers H (2002). The beta-catenin/TCF-4 complex imposes a crypt progenitor phenotype on colorectal cancer cells. Cell..

[12] Duval A, Rolland S, Tubacher E, Bui H, Thomas G, Hamelin R (2000). The human T-cell transcription factor-4 gene: structure, extensive characterization of alternative splicings, and mutational analysis in colorectal cancer cell lines. Cancer Res.

[13] Shang S, Hua F, Hu ZW (2017). The regulation of β-catenin activity and function in cancer: therapeutic opportunities. Oncotarget.

[14] Hsu SM, Raine L, Fanger H (1981). Use of avidin-biotin-peroxidase complex (ABC) in immunoperoxidase techniques: a comparison between ABC and unlabeled antibody (PAP) procedures. J Histochem Cytochem.

[15] Edge S, Byrd DR, Compton CC, Frirz AG, Greene FL, Trotti A (2010). AJCC cancer staging manual.

[16] Sambrook J, Russell DW (2001). Molecular cloning laboratory manual.

[17] Hongyo T, Buzard GS, Calvert RJ, Weghorst CM (1993). 'Cold SSCP': a simple, rapid and non-radioactive method for optimized single-strand conformation polymorphism analyses. Nucleic Acids Res.

[18] Hummon AB, Lim SR, Difilippantonio MJ, Ried T (2007). Isolation and solubilization of proteins after TRIzol extraction of RNA and DNA from patient material following prolonged storage. Biotechniques.

[19] Miyamoto Y, Hosotani R, Wada M, Lee JU, Koshiba T, Fujimoto K (1999). Immunohistochemical analysis of Bcl-2, Bax, Bcl-X, and Mcl-1 expression in pancreatic cancers. Oncology.

[20] Mahmood A, Mahmood S, Desehryver-KecsKimeti K, Alpers DH (1993). Characterization of proteins in rat and human intestinal surfactant particles. Arch Biochem Biophys.

[21] Forrest MP, Hill MJ, Quantock AJ, Martin-Rendon E, Blake DJ (2014). The emerging roles of TCF4 in disease and development. Trends Mol Med.

[22] Shiina H, Igawa M, Breault J, Ribeiro-Filho L, Pookot D, Urakami S, Terashima M, Deguchi M, Yamanaka M, Shirai M, Kaneuchi M, Kane CJ, Dahiya R (2003). The human T-cell factor-4 gene splicing isoforms, Wnt signal pathway, and apoptosis in renal cell carcinoma. Clin Cancer Res.

[23] Duval A, Gayet J, Zhou XP, Iacopetta B, Thomas G, Hamelin R (1999). Frequent frameshift mutations of the TCF-4 gene in colorectal cancers with microsatellite instability. Cancer Res.

[24] Ruckert S, Hiendlmeyer E, Brueckl WM, Oswald U, Beyser K, Dietmaier W (2002). T-cell factor-4 frameshift mutations occur frequently in human microsatellite instability-high colorectal carcinomas but do not contribute to carcinogenesis. Cancer Res.

[25] Jiang Y, Zhou XD, Liu YK, Wu X, Huang XW (2002). Association of hTcf-4 gene expression and mutation with clinicopathological characteristics of hepatocellular carcinoma. World J Gastroenterol.

[26] Cuilliere-Dartigues P, El-Bchiri J, Krimi A, Buhard O, Fontanges P, Fléjou JF (2006). TCF-4 isoforms absent in TCF-4 mutated MSI-H colorectal cancer cells colocalize with nuclear CtBP and repress TCF-4-mediated transcription. Oncogene.

[27] Li CY, Wang Y, Cui ZS, Wang EH (2005). Expression of T cell factor-4 in non-small-cell lung cancer. Chin Med J.

[28] Lin G, Zang T, Zhang Z, Xing D, Guo Y (2000). Expression of T cell factor 4 in renal cell carcinoma. Zhonghua Wai Ke Za Zhi.

[29] Korinek V, Barker N, Moerer P, van Donselaar E, Huls G, Peters PJ, Clevers H (1998). Depletion of epithelial stem-cell compartments in the small intestine of mice lacking Tcf-4. Nat Genet.

[30] Cui J, Zhou X, Liu Y, Tang Z, Romeih M (2003). Wnt signaling in hepatocellular carcinoma: analysis of mutation and expression of beta-catenin, T-cell factor-4 and glycogen synthase kinase 3-beta genes. J Gastroenterol Hepatol.

[31] Rennoll SA, Eshelman MA, Raup-Konsavage WM, Kawasawa YI, Yochum GS. The MYC 3' Wnt-Responsive Element Drives Oncogenic MYC Expression in Human Colorectal Cancer Cells. Cancers (Basel). 2016;8(5). 10.3390/cancers8050052. 10.3390/cancers8050052 PMC4880869 27223305

[32] Zhang X, Ge YL, Tian RH (2009). The knockdown of c-myc expression by RNAi inhibits cell proliferation in human colon cancer HT-29 cells in vitro and in vivo. Cell Mol Biol Lett.

[33] Xu C, Zheng J (2019). siRNA against TSG101 reduces proliferation and induces G0/G1 arrest in renal cell carcinoma - involvement of c-myc, cyclin E1, and CDK2. Cell Mol Biol Lett.

[34] Cadigan KM, Waterman ML. TCF/LEFs and Wnt signaling in the nucleus. Cold Spring Harb Perspect Biol. 2012;4(11). 10.1101/cshperspect.a007906. 10.1101/cshperspect.a007906 PMC3536346 23024173

[35] Takeda K, Kinoshita I, Shimizu Y, Ohba Y, Itoh T, Matsuno Y (2008). Clinicopathological significance of expression of p-c-Jun, TCF4 and beta-catenin in colorectal tumors. BMC Cancer.

[36] Ishiguro H, Wakasugi T, Terashita Y, Sakamoto N, Tanaka T, Sagawa H (2016). Nuclear expression of TCF4/TCF7L2 is correlated with poor prognosis in patients with esophageal squamous cell carcinoma. Cell Mol Biol Lett..

[37] Liu Y, Beyer A, Aebersold R (2016). On the dependency of cellular protein levels on mRNA abundance. Cell.

[38] Plewczyński D, Ginalski K (2009). The interactome: predicting the protein-protein interactions in cells. Cell Mol Biol Lett..

[39] Anwar M, Mahmood S (2019). Colorectal carcinogenesis: a complex malignancy of multiple pathways. EC Gastroenterol Diges Syst.

[40] Forma E, Jóźwiak P, Bryś M, Krześlak A (2014). The potential role of O-GlcNAc modification in cancer epigenetics. Cell Mol Biol Lett.

