# Supplementary material for: TCF 4 tumor suppressor: a molecular target in the prognosis of sporadic colorectal cancer in humans
Source: Cell Mol Biol Lett. 2020 Mar 31;25:24. doi: 10.1186/s11658-020-00217-w (PMC7110825; doi:10.1186/s11658-020-00217-w)
Supplement: Supplementary file 1 — Additional file 1. (PPTX 36670 kb). [file 11658_2020_217_MOESM1_ESM.pptx]

## Slide 1
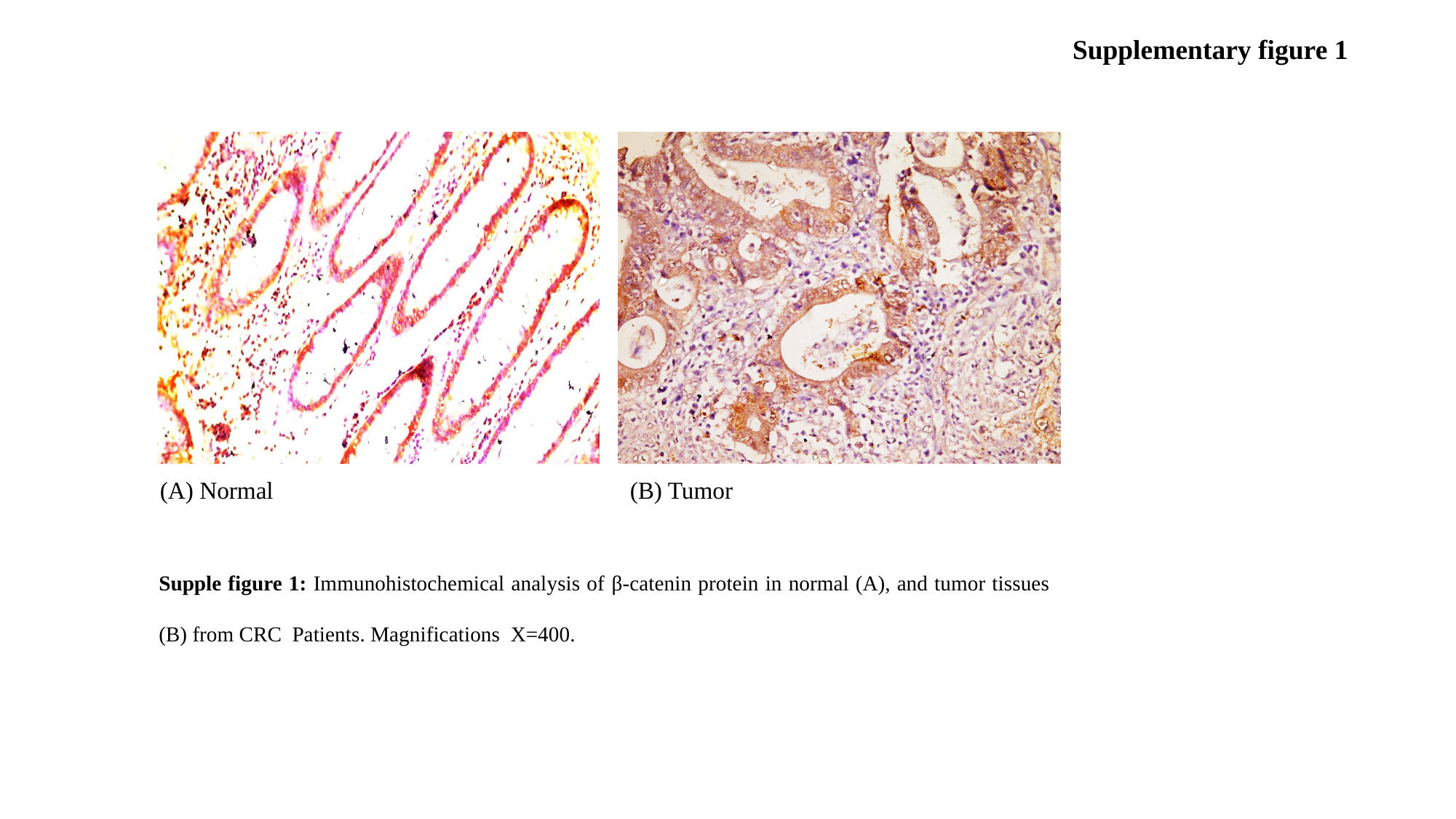

Supplementary figure 1
(A) Normal
(B) Tumor
Supple figure 1: Immunohistochemical analysis of β-catenin protein in normal (A), and tumor tissues (B) from CRC Patients. Magnifications X=400.

## Slide 2
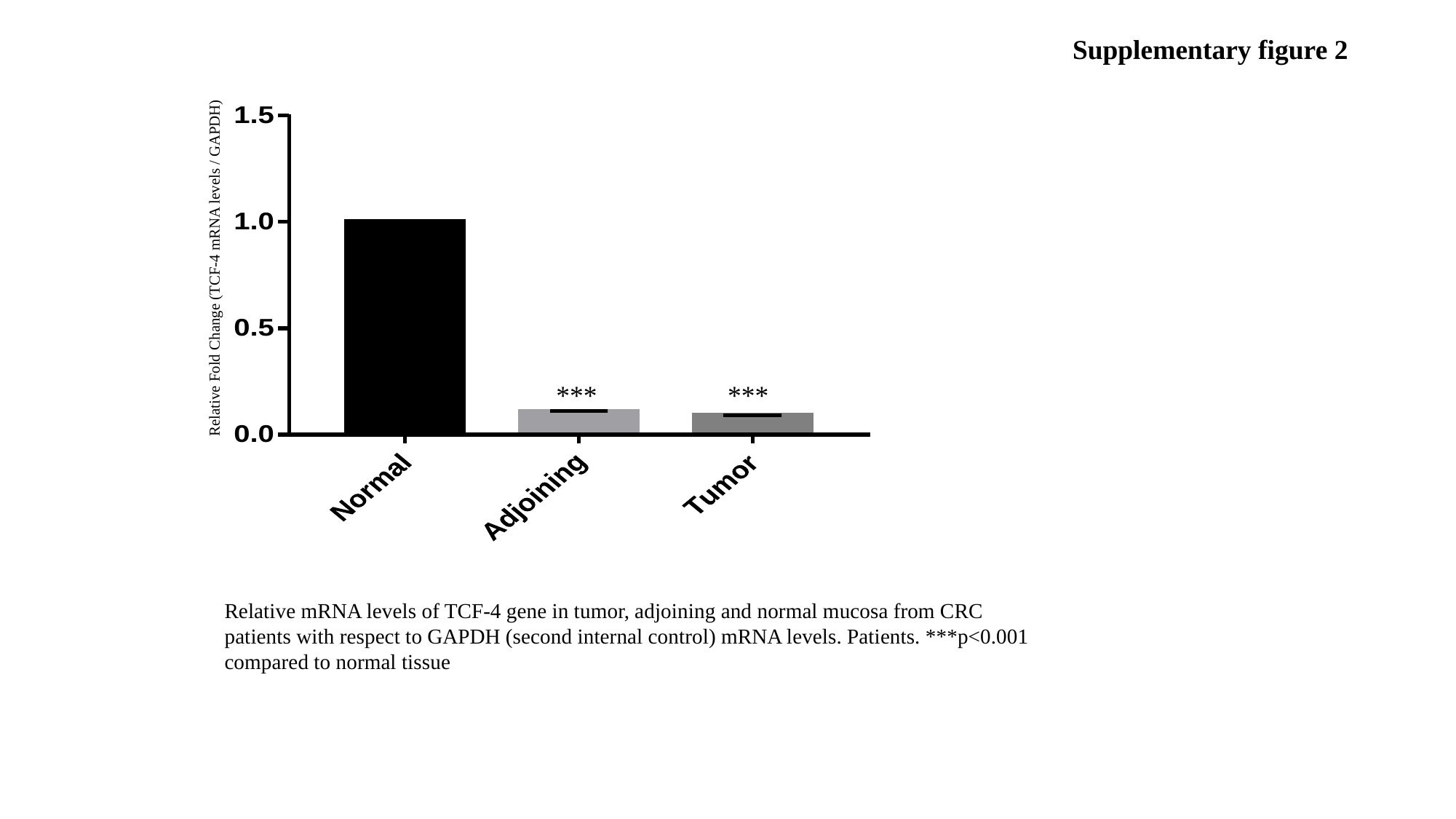

Supplementary figure 2
Relative Fold Change (TCF-4 mRNA levels / GAPDH)
***
***
Relative mRNA levels of TCF-4 gene in tumor, adjoining and normal mucosa from CRC patients with respect to GAPDH (second internal control) mRNA levels. Patients. ***p<0.001 compared to normal tissue
