# Supplementary material for: TCF 4 tumor suppressor: a molecular target in the prognosis of sporadic colorectal cancer in humans
Source: Cell Mol Biol Lett. 2020 Mar 31;25:24. doi: 10.1186/s11658-020-00217-w (PMC7110825; doi:10.1186/s11658-020-00217-w)
Supplement: Supplementary file 2 — Additional file 2. [file 11658_2020_217_MOESM2_ESM.doc]

**Supplementary Table S 1: Comparison of TCF-4 protein expression and TCF-4 gene mutation**

| **Sample No.** | **Tumor** | | | **Adjoining** | | | **Normal** | | |
| --- | --- | --- | --- | --- | --- | --- | --- | --- | --- |
|  | **TCF-4 Protein expression (Nuclear)** | **TCF-4 Protein expression (Cytoplasmic)** | **TCF-4 Tumor Mutation** | **TCF-4 Protein expression (Nuclear)** | **TCF-4 Protein expression (Cytoplasmic)** | **TCF-4 Adjoining Mutation** | **TCF-4 Protein expression (Nuclear)** | **TCF-4 Protein expression (Cytoplasmic)** | **TCF-4 Mutation** |
| S1 | + | + | ‒ | + | + | ‒ | + | + | ‒ |
| S2 | + | + | + | + | + | ‒ | + | + | ‒ |
| S3 | ‒ | + | ‒ | ‒ | + | ‒ | ‒ | + | ‒ |
| S4 | + | + | ‒ | + | + | ‒ | + | + | ‒ |
| S5 | + | + | ‒ | + | + | ‒ | + | + | ‒ |
| S6 | + | + | ‒ | + | + | ‒ | + | + | ‒ |
| S7 | + | + | ‒ | + | + | ‒ | + | + | ‒ |
| S8 | ‒ | + | ‒ | ‒ | + | ‒ | ‒ | + | ‒ |
| S9 | + | + | ‒ | + | + | ‒ | + | + | ‒ |
| S10 | + | + | ‒ | + | + | ‒ | + | + | ‒ |
| S11 | + | + | + | + | + | ‒ | + | + | ‒ |
| S12 | + | ‒ | ‒ | + | ‒ | ‒ | + | ‒ | ‒ |
| S13 | + | + | ‒ | + | + | ‒ | + | + | ‒ |
| S14 | + | + | ‒ | + | + | ‒ | + | + | ‒ |
| S15 | + | ‒ | ‒ | + | ‒ | ‒ | + | ‒ | ‒ |
| S16 | + | ‒ | ‒ | + | ‒ | ‒ | + | ‒ | ‒ |
| S17 | + | + | ‒ | + | + | ‒ | + | + | ‒ |
| S18 | + | ‒ | ‒ | + | ‒ | ‒ | + | ‒ | ‒ |
| S19 | + | ‒ | ‒ | + | ‒ | ‒ | + | ‒ | ‒ |
| S20 | + | + | ‒ | + | + | ‒ | + | + | ‒ |
| S21 | + | + | ‒ | + | + | ‒ | + | + | ‒ |
| S22 | + | ‒ | ‒ | + | ‒ | ‒ | + | ‒ | ‒ |
| S23 | + | ‒ | ‒ | + | ‒ | ‒ | + | ‒ | ‒ |
| S24 | + | ‒ | ‒ | + | ‒ | ‒ | + | ‒ | ‒ |
| S25 | + | + | ‒ | + | + | ‒ | + | + | ‒ |
| S26 | + | ‒ | ‒ | + | ‒ | ‒ | + | ‒ | ‒ |
| S27 | + | ‒ | ‒ | + | ‒ | ‒ | + | ‒ | ‒ |
| S28 | + | + | ‒ | + | + | ‒ | + | + | ‒ |
| S29 | + | + | ‒ | + | + | ‒ | + | + | ‒ |
| S30 | + | + | ‒ | + | + | ‒ | + | + | ‒ |
| S31 | + | + | ‒ | + | + | ‒ | + | + | ‒ |
| S32 | ‒ | + | ‒ | ‒ | + | ‒ | ‒ | + | ‒ |
| S33 | + | + | + | + | + | ‒ | + | + | ‒ |
| S34 | + | + | + | + | + | ‒ | + | + | ‒ |
| S35 | ‒ | + | ‒ | ‒ | + | ‒ | ‒ | + | ‒ |
| S36 | + | + | ‒ | ‒ | + | ‒ | ‒ | + | ‒ |
| S37 | + | + | ‒ | + | + | ‒ | + | + | ‒ |
| S38 | + | + | ‒ | + | + | ‒ | + | + | ‒ |
| S39 | + | + | ‒ | + | + | ‒ | + | + | ‒ |
| S40 | ‒ | + | ‒ | ‒ | + | ‒ | ‒ | + | ‒ |
| S41 | + | + | ‒ | + | + | ‒ | + | + | ‒ |
| S42 | + | + | ‒ | + | + | ‒ | + | + | ‒ |
| S43 | + | + | ‒ | ‒ | + | ‒ | ‒ | + | ‒ |
| S44 | ‒ | + | ‒ | ‒ | + | ‒ | ‒ | + | ‒ |
| S45 | + | + | ‒ | + | + | ‒ | + | + | ‒ |
| S46 | ‒ | + | ‒ | ‒ | + | ‒ | ‒ | + | ‒ |
| S47 | ‒ | + | ‒ | ‒ | + | ‒ | ‒ | + | ‒ |
| S48 | + | + | ‒ | + | + | ‒ | + | + | ‒ |
| S49 | + | + | ‒ | + | + | ‒ | + | + | ‒ |
| S50 | + | + | + | + | + | ‒ | + | + | ‒ |
| S51 | + | + | ‒ | ‒ | + | ‒ | ‒ | + | ‒ |
| S52 | ‒ | + | ‒ | ‒ | + | ‒ | ‒ | + | ‒ |
| S53 | + | + | ‒ | + | + | ‒ | + | + | ‒ |
| S54 | + | + | ‒ | + | + | ‒ | + | + | ‒ |
| S55 | + | + | ‒ | + | + | ‒ | + | + | ‒ |
| S56 | ‒ | + | ‒ | ‒ | + | ‒ | ‒ | + | ‒ |
| S57 | ‒ | + | ‒ | ‒ | + | ‒ | ‒ | + | ‒ |
| S58 | + | + | ‒ | + | + | ‒ | + | + | ‒ |
| S59 | + | + | ‒ | + | + | ‒ | + | + | ‒ |
| S60 | + | + | ‒ | + | + | ‒ | + | + | ‒ |
